# *Ishige foliacea* Water Extract (KDC22-01) Attenuates Tau Hyperphosphorylation and Ameliorates Scopolamine-Induced Memory Impairment in Mice

**DOI:** 10.3390/nu18213517

**Published:** 2026-10-26

**Authors:** Jung Ho Choi, Won Jin Baek, Su-Hyun Kim, Myoung-Sook Shin

**Affiliations:** 1 R&D Center Pharmaceutical Laboratory, Korean Drug Co., Ltd., Seoul 06300, Republic of Korea; jhchoi@nicepharma.com; 2 Department of Beauty Science, Kwangju Women’s University, Kwangju 62396, Republic of Korea; 3 Department of Beauty Art, Youngsan University, Busan 48015, Republic of Korea; 4 College of Korean Medicine, Gachon University, Seongnam 13120, Republic of Korea

**Keywords:** *Ishige foliacea*, KDC22-01, tau hyperphosphorylation, scopolamine, memory impairment, neuroprotection

## Abstract

**Background/Objectives**: *Ishige foliacea* is an edible brown alga containing bioactive polyphenols with reported neuroprotective properties. Although extracts of *I. foliacea* have previously been shown to ameliorate cognitive impairment, their effects on pathological tau phosphorylation remain poorly characterized. This study investigated the neuroprotective and cognition-modulating effects of KDC22-01, an aqueous extract of *I. foliacea*. **Methods**: KDC22-01 was chemically characterized by total polyphenol and flavonoid-equivalent contents and HPLC fingerprinting. Its effects on pre-aggregated Aβ25–35-induced cytotoxicity and okadaic acid (OA)-induced tau phosphorylation were evaluated in SH-SY5Y cells. Cognitive performance and hippocampal tau phosphorylation were further assessed in male C57BL/6 mice with scopolamine (SCO)-induced cognitive impairment following oral KDC22-01 administration (100 or 300 mg/kg). **Results**: KDC22-01 protected SH-SY5Y cells against Aβ25–35-induced cytotoxicity, restoring cell viability from approximately 59% to 87% of control levels at 50 μg/mL. KDC22-01 also reduced the OA-induced *p*-tau (Ser199)/total tau ratio at 25 and 50 μg/mL. In SCO-treated mice, 100 mg/kg KDC22-01 significantly improved performance in the Morris water maze and passive avoidance test, whereas improvements in Y-maze spontaneous alternation did not reach statistical significance. Both KDC22-01 doses reduced hippocampal *p*-tau (Ser396), while changes in hippocampal Aβ levels were not statistically significant. **Conclusions**: These findings suggest that attenuation of tau hyperphosphorylation may contribute to the cognitive effects of KDC22-01 and support further investigation of this aqueous *I. foliacea* extract as a candidate functional-food ingredient for cognitive health.

## 1. Introduction

Alzheimer’s disease (AD) is a progressive neurodegenerative disorder and the leading cause of dementia, characterized biologically by amyloid-β (Aβ) deposition and tau pathology and clinically by progressive impairment of memory and other cognitive functions [1]. In addition to amyloid and tau abnormalities, dysfunction of the basal forebrain cholinergic system is closely associated with cognitive decline because cholinergic projections to the cerebral cortex and hippocampus critically regulate attention, learning, and memory [2,3]. Basal forebrain cholinergic neurons are selectively vulnerable during aging and AD, and their degeneration contributes to impaired cholinergic neurotransmission and cognitive dysfunction [2,3,4]. Tau, a microtubule-associated protein involved in cytoskeletal stabilization and axonal function, undergoes abnormal hyperphosphorylation in AD, reducing its affinity for microtubules and promoting the formation of paired helical filaments and neurofibrillary tangles [5]. Importantly, increased cerebral tau burden is strongly associated with subsequent cognitive deterioration, and tau-positron emission tomography measures have shown substantial prognostic value for predicting cognitive decline in individuals across the symptomatic AD continuum [6]. These observations support both cholinergic dysfunction and abnormal tau phosphorylation as biologically relevant targets for investigating interventions aimed at preserving cognitive function [4,5,6].

Scopolamine, a non-selective muscarinic acetylcholine receptor antagonist, produces reproducible deficits in learning, memory, and attention and is therefore widely used as a pharmacological model of cholinergic cognitive impairment in the preclinical evaluation of cognition-modulating agents [7,8]. However, scopolamine produces an acute and reversible cholinergic deficit and does not reproduce the progressive and multifactorial neuropathology of AD. Therefore, it is more appropriately regarded as a model of cholinergic cognitive impairment rather than a complete model of AD [7,8]. At the cellular level, okadaic acid (OA)-mediated inhibition of serine/threonine protein phosphatases is commonly used to induce tau hyperphosphorylation in SH-SY5Y cells, providing a complementary experimental system for evaluating compounds that modulate pathological tau phosphorylation [9,10]. The combined use of pharmacological models targeting cholinergic dysfunction and tau phosphorylation can therefore provide complementary information on the cognitive and molecular effects of candidate neuroprotective agents [7,9].

Marine brown algae are rich sources of structurally diverse bioactive metabolites, particularly phlorotannins, a class of phloroglucinol-based polyphenols characteristic of brown algae that have attracted considerable interest for their antioxidant and neuroprotective properties [11,12]. Experimental studies have indicated that phlorotannin-rich extracts and individual phlorotannins, including dieckol and related compounds, can protect neuronal cells from oxidative or toxic injury and modulate pathways associated with cholinergic function, oxidative stress, neuroinflammation, and cognitive impairment [12,13]. *Ishige foliacea*, an edible brown alga used as a functional food in East Asian countries, has attracted attention as a source of biologically active phenolic compounds [14]. A previous study demonstrated that an ethanol extract of *I. foliacea* exhibited antioxidant and acetylcholinesterase- and β-secretase-inhibitory activities, protected SH-SY5Y cells against H_2_O_2_- and Aβ-induced cytotoxicity, and ameliorated scopolamine-induced memory deficits by modulating hippocampal BDNF–TrkB–ERK signaling [14]. The study focused primarily on oxidative stress, amyloid-related enzyme activities, cholinergic mechanisms, and neurotrophic signaling [5,14]; the effect of *I. foliacea* on pathological tau phosphorylation remains insufficiently characterized. In contrast to the ethanol extract used in that study, we prepared an aqueous extract, because water extraction avoids organic solvent residues and is suitable for manufacturing food-grade ingredients. The present study investigated the neuroprotective and cognition-modulating effects of an aqueous extract of *I. foliacea* (termed KDC22-01) by evaluating Aβ25–35-induced cytotoxicity and OA-induced tau hyperphosphorylation in SH-SY5Y cells and by assessing cognitive performance and hippocampal tau phosphorylation in a scopolamine-induced mouse model of cognitive impairment [7,9,14]. Therefore, the present study focused on the effects of an aqueous *I. foliacea* extract (KDC22-01) on Aβ-induced neuronal injury and tau hyperphosphorylation in vitro and on cognitive performance and hippocampal tau phosphorylation in a scopolamine-induced model of cholinergic cognitive impairment.

## 2. Materials and Methods

### 2.1. Preparation and Characterization of KDC22-01

Dried *I. foliacea* collected on Chuja Island, Korea on June, 2022, was purchased from PARA Jeju (Jeju Island, Republic of Korea). Twenty-one kilograms of dried *I. foliacea* was extracted with 100% distilled water at 70 °C for 4 h. The extract was filtered through a 10-μm filter and concentrated to 13.91 kg. The concentrate was pasteurized at 90 °C for 20 min, then spray-dried (inlet temperature, 180–200 °C; outlet temperature, 81–87 °C) without addition of excipients, yielding 144 g of KDC22-01.

The chromatographic fingerprint of KDC22-01 was characterized using reversed-phase high-performance liquid chromatography, with dieckol identified as the marker phlorotannin. In brief, the extract was dissolved in methanol at approximately 10 mg/mL, filtered through a 0.45-μm polyvinylidene difluoride (PVDF) syringe filter, and injected (10 μL) into a C18 column (4.6 × 250 mm, 4 μm) maintained at 25 °C. The mobile phase consisted of 0.1% phosphoric acid in water (A) and 0.1% phosphoric acid in acetonitrile (B), delivered isocratically at 75:25 (A:B, *v*/*v*) for 25 min at a flow rate of 1.0 mL/min, with UV detection at 230 nm. A representative chromatogram is presented in Supplementary Figure S1.

### 2.2. Quantification of Total Polyphenols and Total Flavonoids

The total polyphenol content of KDC22-01 was determined using the Folin–Ciocalteu method [15]. KDC22-01 was dissolved in 50% (*v*/*v*) aqueous methanol at 1 mg/mL, and 0.5 mL of the sample was mixed with 2.5 mL of 10-fold diluted Folin–Ciocalteu reagent (Sigma-Aldrich, St. Louis, MO, USA). After incubation at room temperature for 5 min, 2 mL of 7.5% (*w*/*v*) sodium carbonate was added, and the mixture was incubated in the dark for 60 min. The absorbance at 765 nm was measured using a UV-visible spectrophotometer, and the total polyphenol content was calculated from a gallic acid calibration curve (10–200 μg/mL; r^2^ > 0.999) and expressed as gallic acid equivalents (mg GAE/g extract). The total flavonoid content was determined using the aluminum chloride colorimetric method [16]. In brief, 0.5 mL of sample solution (1 mg/mL in methanol) was mixed with 1.5 mL of methanol, 0.1 mL of 10% (*w*/*v*) aluminum chloride, 0.1 mL of 1 M potassium acetate, and 2.8 mL of distilled water. After 30 min of incubation at room temperature, the absorbance at 415 nm was measured. The total flavonoid content was calculated from a quercetin calibration curve (10–100 μg/mL; r^2^ > 0.999) and expressed as quercetin equivalents (mg QE/g extract). All measurements were performed in triplicate.

### 2.3. In Vitro Experiments

#### 2.3.1. Cell Culture

SH-SY5Y human neuroblastoma cells (KCLB No. 22266; RRID: CVCL_0019) were obtained from the Korean Cell Line Bank (KCLB, Seoul, Republic of Korea) and cultured in Dulbecco’s modified Eagle’s medium (Gibco/Thermo Fisher Scientific, Waltham, MA, USA) supplemented with 10% fetal bovine serum (Corning, Corning, NY, USA) and 1% penicillin-streptomycin (Gibco/Thermo Fisher Scientific) at 37 °C in a humidified atmosphere containing 5% CO_2_.

#### 2.3.2. Cytotoxicity and Cytoprotective Assays

To evaluate the cytotoxicity of KDC22-01, SH-SY5Y cells were treated with KDC22-01 at final concentrations of 5, 10, 25, 50, or 100 μg/mL for 24 h, and cell viability was assessed using the 3-(4,5-dimethylthiazol-2-yl)-2,5-diphenyltetrazolium bromide (MTT) assay.

For the cytoprotective assay, cells were pretreated with KDC22-01 (25 and 50 μg/mL) for 24 h. After 24 h of pretreatment, pre-aggregated Aβ25–35 (final concentration of 50 μM) was added to the culture medium without removing KDC22-01, and the cells were incubated for an additional 48 h. Pre-aggregated Aβ25–35 was prepared by dissolving the peptide in sterile double-distilled water at a concentration of 1 mM and incubating the solution at 37 °C for 7 days prior to use [17,18,19]. The 48-h exposure was chosen because Aβ25–35-induced cytotoxicity develops gradually through oxidative stress and apoptosis [17,20]. Following treatment, MTT was added to each well at a final concentration of 0.5 mg/mL, and the cells were incubated at 37 °C for 4 h. Then, the medium was carefully removed, and 100 μL of dimethyl sulfoxide was added to each well to dissolve the formed formazan crystals. The absorbance at 570 nm was measured using a Synergy HT microplate reader (BioTek Instruments, Winooski, VT, USA). Each condition was tested in three replicate wells, and the experiment was independently repeated three times. To characterize the concentration-dependent cytotoxicity of Aβ25–35 and OA, cells were exposed to pre-aggregated Aβ25–35 (10, 50, 100, or 200 μM; prepared as described above) for 48 h or to OA (10, 30, 60, or 90 nM) for 12 h, and cell viability was assessed using the MTT assay.

#### 2.3.3. Quantification of Tau Hyperphosphorylation

To assess the attenuation of OA-induced tau hyperphosphorylation by KDC22-01, SH-SY5Y cells were seeded in six-well plates at 1.0 × 10^6^ cells/well and cultured for 24 h. Then, the cells were pretreated with KDC22-01 at concentrations of 25 and 50 μg/mL for 12 h. Subsequently, OA was added to the culture medium at a final concentration of 60 nM without removing KDC22-01. After incubation for an additional 12 h to induce tau hyperphosphorylation, the cells were harvested and lysed in ice-cold lysis buffer supplemented with protease and phosphatase inhibitors [9,10]. A short OA exposure was used because OA increases tau phosphorylation within hours by inhibiting PP2A, whereas longer exposure causes cell death that would confound tau measurements [21,22]. The 12-h pretreatment made the total exposure to KDC22-01 (24 h) the same as in the cytotoxicity assay. Total tau and tau phosphorylated at Ser199 in the lysates were quantified using a Human Tau ELISA Kit (ab273617, Abcam, Cambridge, UK) and a Human Phospho-Tau (Ser199) ELISA Kit (KHB7041; Invitrogen, Thermo Fisher Scientific, Waltham, MA, USA), respectively, according to the manufacturers’ instructions. Absorbance was measured at 450 nm using a Synergy HT microplate reader. Tau phosphorylation was expressed as the ratio of *p*-tau Ser199 to total tau, normalized to the mean of the control group. Ser199 was selected because it lies in the proline-rich region of tau, is phosphorylated by GSK-3β and CDK5 and dephosphorylated by PP2A and therefore responds directly to OA-induced PP2A inhibition [5,23].

### 2.4. Animal Experiments

All animal experiments were conducted at the Laboratory Animal Research Center of Dankook University in accordance with the ARRIVE guidelines [24], and the protocol was approved by the Institutional Animal Care and Use Committee of Dankook University (protocol code DKU-22-073, approved on 4 October 2022).

Four-week-old male C57BL/6 mice (KOATECH Animal, Pyeongtaek, Korea) were acclimatized to the laboratory environment (humidity controlled at 55% ± 5% and temperature at 24 ± 1 °C, with a 12-h/12-h night/day cycle) for one week prior to the experiments. The mice received a chow diet and water ad libitum. After the one-week adaptation period, mice were randomly assigned into four groups (*n* = 6 per group, total 24 mice) using a random number generator: a control group (vehicle only), a scopolamine (SCO) group (SCO + vehicle), a KDC22-01 100 mg/kg group (SCO + KDC22-01 100 mg/kg), and a KDC22-01 300 mg/kg group (SCO + KDC22-01 300 mg/kg). Because this was the first in vivo evaluation of KDC22-01, the doses were selected on the basis of its intended daily intake as a health functional food. Using body surface area-based conversion (Km of 3 for mice and 37 for humans) [25], 100 and 300 mg/kg correspond to human equivalent doses of approximately 8.1 and 24.3 mg/kg, or 0.49 and 1.46 g/day for a 60-kg adult. These doses are within the range used in previous in vivo studies of *I. foliacea* preparations (50–500 mg/kg) [26,27], and a three-fold interval was used to examine dose dependence. The experimental schedule is added. KDC22-01 (100 or 300 mg/kg) or vehicle was administered orally by gavage once daily for 12 consecutive days. After the 12-day pretreatment period, behavioral tests (Y-maze, passive avoidance and Morris water maze tests) were performed on days 13–15, one test per day. KDC22-01 was not administered on the test days. Cognitive impairment was induced by intraperitoneal injection of SCO (1.4 mg/kg, dissolved in water) 30 min before each test, and the control group received the vehicle. All behavioral testing was performed by an experimenter blinded to group allocation. After the final behavioral test, mice were deeply anesthetized with isoflurane and euthanized by cervical dislocation. The hippocampi were then dissected and stored at −80 °C until Western blot analysis [8,28,29].

#### 2.4.1. Morris Water Maze Test

The Morris water maze test was used to assess spatial learning and memory. The test was carried out in a circular pool (diameter: 100 cm; height: 50 cm) filled with water maintained at 25 ± 1 °C. Spatial cues were maintained at fixed positions around the pool, and a circular escape platform (diameter: 5 cm) was submerged 1 cm below the water surface. All mice underwent two training trials followed by one test trial. During each trial, mice were allowed a maximum of 180 s to locate the submerged platform, and escape latency was recorded. Behavioral data were analyzed using EthoVision XT software (version 15.0; Noldus Information Technology, Wageningen, The Netherlands).

#### 2.4.2. Passive Avoidance Test

The passive avoidance test was used to assess learning-associated long-term memory. The apparatus consisted of interconnected light and dark compartments, with a heat block installed in the dark compartment to provide an aversive thermal stimulus. During the training trial, each mouse was placed in the light compartment and allowed to enter the dark compartment according to its natural preference, where an aversive thermal stimulus was applied. Following a single training trial, retention memory was evaluated by measuring the duration of time that the mouse remained in the light compartment during the test trial.

#### 2.4.3. Y-Maze Test

The Y-maze test was used to assess short-term spatial working memory. The apparatus consisted of three identical arms (30 cm long × 5 cm wide × 15 cm high) arranged at 120° angles. Each mouse was placed at the end of one arm and allowed to explore freely for 5 min. Arm entries were recorded when all four paws crossed into an arm. Spontaneous alternation was defined as consecutive entries into all three arms (e.g., ABC, BCA, CAB) and was calculated as:

Alternation (%) = (number of alternations/[total arm entries − 2]) × 100

### 2.5. Western Blot Analysis

Hippocampal tissues were homogenized in ice-cold radioimmunoprecipitation assay buffer containing a protease and phosphatase inhibitor cocktail and centrifuged at 12,000× *g* and 4 °C for 20 min. Protein concentrations in the supernatants were determined using the bicinchoninic acid assay (Thermo Fisher Scientific). Equal amounts of protein (20 μg) were resolved through 10–16% sodium dodecyl sulfate-polyacrylamide gel electrophoresis and transferred to Immobilon-P PVDF membranes (Millipore, Billerica, MA, USA). The membranes were blocked in 5% skim milk in Tris-buffered saline with Tween-20 (TBST) at room temperature for 2 h and probed overnight at 4 °C with the following primary antibodies: anti-tau (#46687, rabbit monoclonal, 1:1000 dilution), anti-phospho-tau Ser396 (#9632, mouse monoclonal, 1:1000 dilution), and anti-β-actin (#3700, mouse monoclonal, 1:5000 dilution) (all from Cell Signaling Technology, Danvers, MA, USA). Aβ1–42 was detected using a rabbit monoclonal antibody (D9A3A, #14974; Cell Signaling Technology). β-Actin was used as a loading control. After five washes in TBST (10 min each), the membranes were incubated with horseradish peroxidase-conjugated anti-rabbit or anti-mouse IgG secondary antibodies (1:10,000) at room temperature for 1 h. Bands were visualized with enhanced chemiluminescence reagent (Millipore Sigma) and imaged using a ChemiDoc XRS system (Bio-Rad, Hercules, CA, USA). Band intensities were quantified using the ImageJ software ver1.54. (NIH, Bethesda, MD, USA).

### 2.6. Statistical Analysis

All data are presented as mean ± standard deviation (SD). For single-time-point comparisons among multiple groups, including the MTT assay, tau ELISA, Y-maze test, passive avoidance test, probe-trial outcomes, and Western blot analyses, data were analyzed via one-way analysis of variance (ANOVA) followed by Tukey’s multiple-comparison test. Statistical analyses were performed using the SPSS software (version 25.0; IBM, Armonk, NY, USA).

## 3. Results and Discussion

### 3.1. Chemical Characterization of KDC22-01

KDC22-01 contained 54.06 ± 2.24 mg GAE/g extract and 43.26 ± 0.71 mg QE/g extract, as determined using the Folin–Ciocalteu and aluminum chloride colorimetric methods, respectively, with gallic acid and quercetin as external standards. Because the same batch of KDC22-01 was used in all experiments, the amounts of total polyphenols delivered were 1.35 and 2.70 µg GAE/mL at 25 and 50 µg/mL in vitro and 5.41 and 16.2 mg GAE/kg at 100 and 300 mg/kg in vivo.

### 3.2. In Vitro Neuroprotective Effects of KDC22-01

#### 3.2.1. KDC22-01 Protects Against Aβ25–35-Induced Toxicity in SH-SY5Y Cells

**Figure 1:**
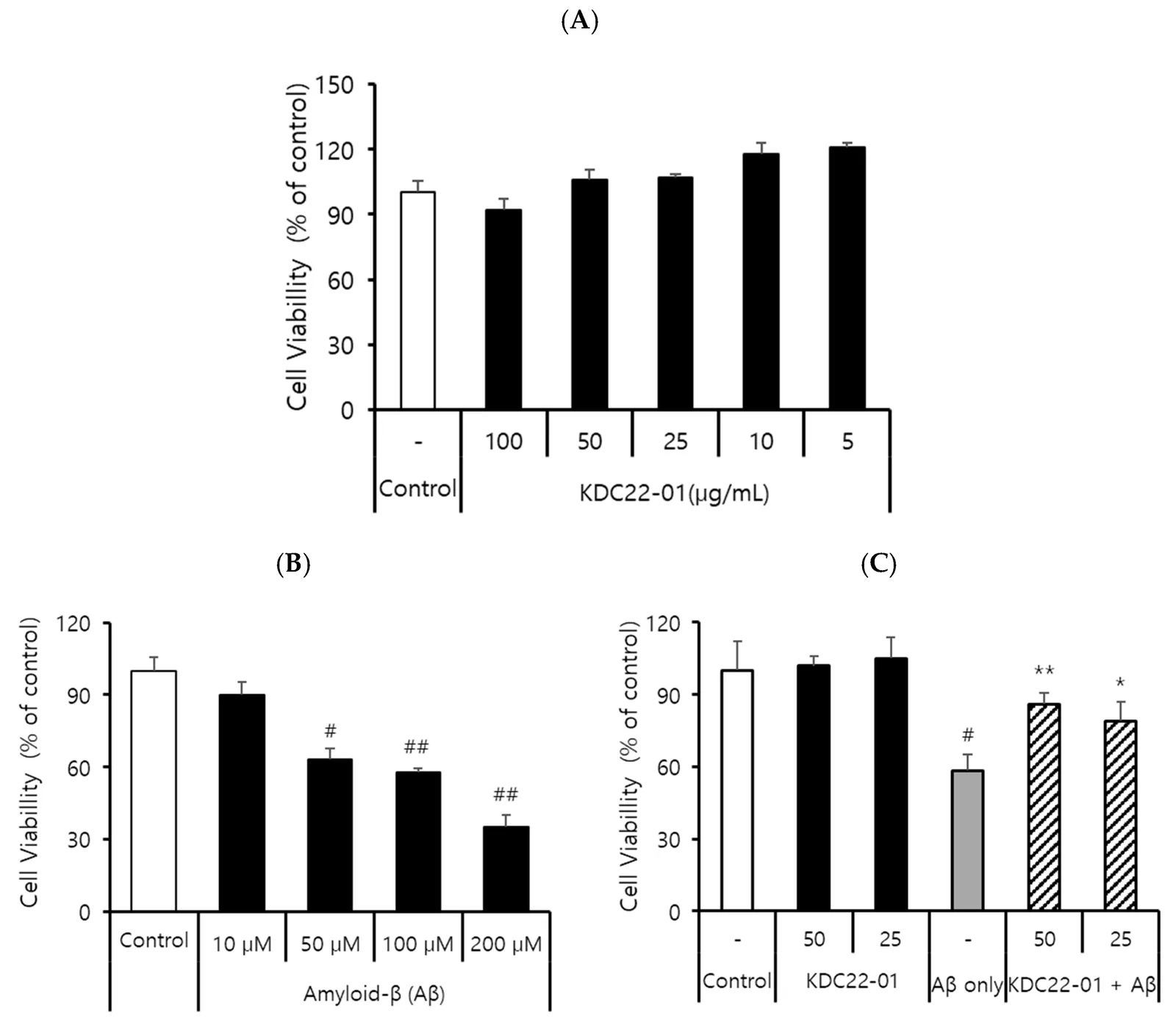
KDC22-01 protects SH-SY5Y cells against Aβ25–35-induced toxicity. (**A**) Cell viability of SH-SY5Y cells treated with KDC22-01 at 5–100 μg/mL for 24 h. (**B**) Cell viability of SH-SY5Y cells exposed to pre-aggregated Aβ25–35 at 10–200 μM for 48 h. (**C**) Protective effect of KDC22-01 (25 or 50 μg/mL) on pre-aggregated Aβ25–35 (50 μM)-induced cytotoxicity. Cells were pretreated with KDC22-01 for 24 h, then exposed to Aβ25–35 for 48 h in the continued presence of KDC22-01. Cell viability was measured using the MTT assay and is expressed as a percentage of the control. Data are presented as the mean ± SD of three independent experiments. # *p* < 0.01 and ## *p* < 0.001 vs. control; * *p* < 0.01 and ** *p* < 0.001 vs. Aβ25–35.

To identify non-cytotoxic concentrations of KDC22-01, SH-SY5Y human neuroblastoma cells were treated with 5–100 μg/mL KDC22-01 for 24 h. KDC22-01 did not significantly reduce cell viability at any of the concentrations tested (Figure 1A). Based on these results, 25 and 50 μg/mL were used in subsequent cell experiments. Pre-aggregated Aβ25–35 decreased cell viability in a concentration-dependent manner, to approximately 90%, 75%, 58%, and 35% of the control at 10, 50, 100, and 200 μM, respectively (Figure 1B). Thus, 50 μM, the concentration used in the cytoprotection assay, caused a moderate, submaximal loss of viability, which allows protective effects to be detected.

In the cytoprotection assay, exposure to pre-aggregated Aβ25–35 (50 μM) for 48 h significantly reduced cell viability compared with the no-treatment control, whereas KDC22-01 alone (25 or 50 μg/mL) did not affect cell viability. Pretreatment with KDC22-01 at 25 or 50 μg/mL significantly attenuated Aβ25–35-induced loss of cell viability, particularly at 50 μg/mL (Figure 1C). These results indicate that KDC22-01 exerts a cytoprotective effect against Aβ25–35-induced toxicity within the concentration range tested. This finding is consistent with previous studies showing that Aβ25–35 induces marked cytotoxic and apoptotic responses in neuronal cell models and that natural polyphenolic compounds or extracts can attenuate Aβ25–35-induced injury in SH-SY5Y cells [17,18,19,20].

#### 3.2.2. KDC22-01 Attenuates OA-Induced Tau Hyperphosphorylation

**Figure 2:**
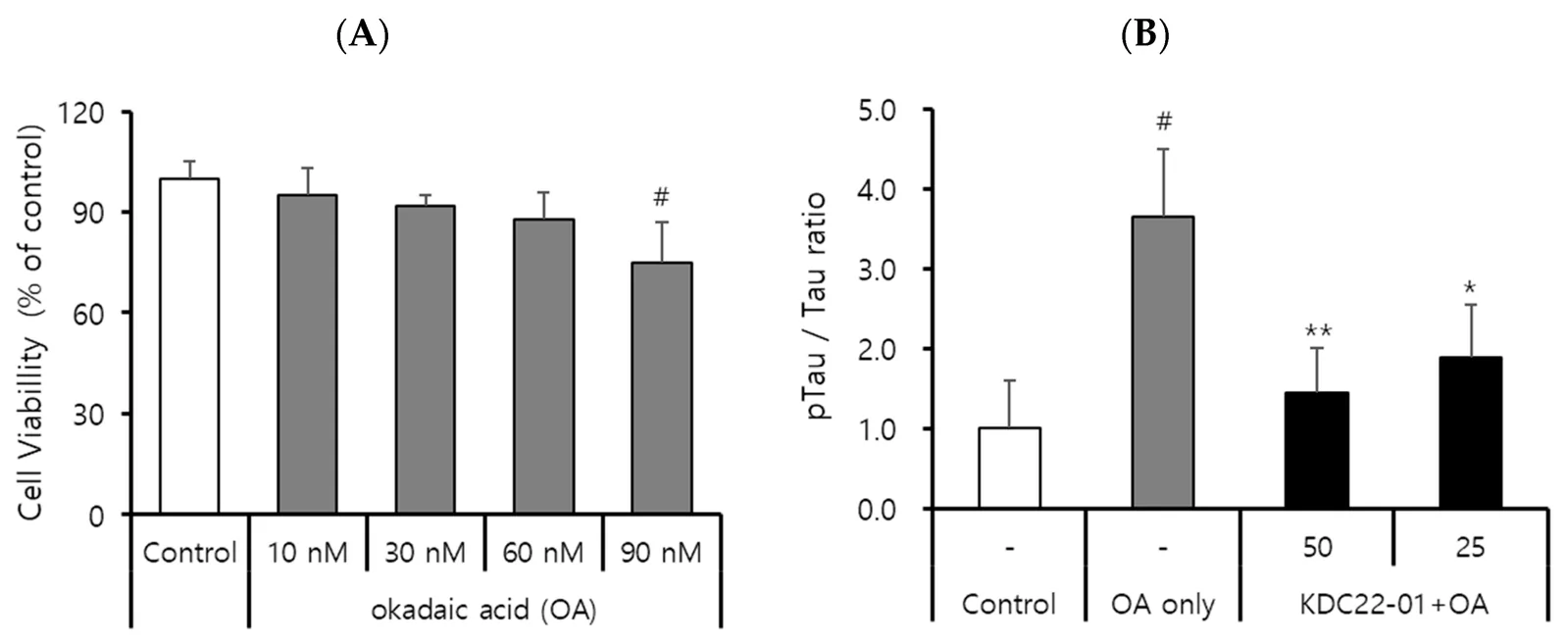
KDC22-01 attenuates okadaic acid (OA)-induced tau hyperphosphorylation in SH-SY5Y cells. (**A**) Cell viability of SH-SY5Y cells exposed to OA at 10–90 nM for 12 h, measured using the MTT assay and expressed as a percentage of the control. (**B**) SH-SY5Y cells were treated with KDC22-01 (25 or 50 μg/mL) for 12 h. Then, OA (60 nM) was added directly to the KDC22-01-containing medium, without washing or medium replacement. The cells were incubated for an additional 12 h, resulting in total exposure times of 24 h for KDC22-01 and 12 h for OA. Total tau and *p*-tau (Ser199) were quantified using ELISA kits, and tau phosphorylation is expressed as the *p*-tau (Ser199)/total tau ratio. Data are presented as the mean ± SD of three independent experiments. In (**A**), # *p* < 0.05 vs. control; in (**B**), # *p* < 0.001 vs. control and * *p* < 0.01, ** *p* < 0.001 vs. OA.

Before examining tau phosphorylation, we assessed the effect of OA alone on cell viability. OA decreased cell viability in a concentration-dependent manner, but the decrease was significant only at 90 nM (approximately 75% of the control; Figure 2A). At 60 nM, the concentration used to induce tau hyperphosphorylation, viability remained at approximately 88% of the control, with no significant difference. Therefore, the changes in tau phosphorylation at this concentration are unlikely to be attributable to cytotoxicity.

We next examined whether KDC22-01 could attenuate tau phosphorylation induced by OA exposure. SH-SY5Y cells were exposed to KDC22-01 (25 or 50 μg/mL) for 12 h, then OA (60 nM) was added directly to the KDC22-01-containing medium and incubation was continued for an additional 12 h. OA treatment markedly increased the *p*-tau (Ser199)/total tau ratio compared with the no-treatment control (Figure 2B). KDC22-01 significantly reduced the OA-induced increase in the *p*-tau (Ser199)/total tau ratio at both 25 and 50 μg/mL. These findings indicate that KDC22-01 attenuates OA-induced tau phosphorylation while OA remains present in the culture medium. OA-induced tau hyperphosphorylation is primarily due to the inhibition of serine/threonine phosphatases, particularly PP2A-dependent tau dephosphorylation, whereas GSK-3β and other tau kinases also contribute to balance tau phosphorylation. Hence, the reduced *p*-tau/total tau ratio after KDC22-01 treatment suggests that KDC22-01 may modulate kinase–phosphatase signaling involved in tau phosphorylation.

### 3.3. In Vivo Effects of KDC22-01 on SCO-Induced Memory Impairment

**Figure 3:**
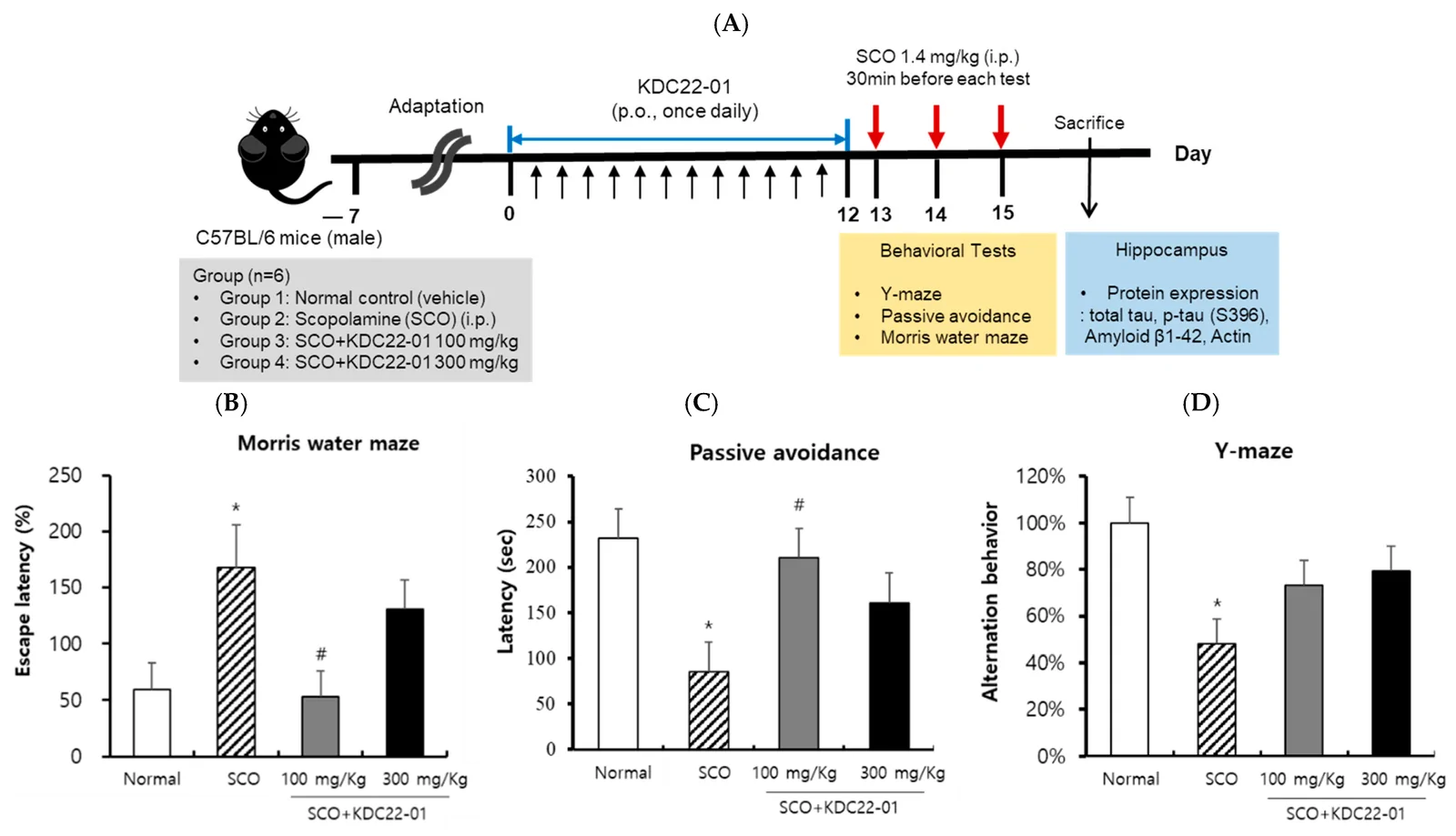
KDC22-01 ameliorates cognitive function in scopolamine (SCO)-treated mice. (**A**) Experimental design. After 1 week of adaptation, mice received KDC22-01 (100 or 300 mg/kg) or vehicle orally once daily for 12 days. Behavioral tests were performed on days 13–15, with SCO (1.4 mg/kg, i.p.) administered 30 min before each test. After the final test, the hippocampi were collected for Western blot analysis of total tau, *p*-tau (Ser396), Aβ1–42 and β-actin. (**B**) Escape latency in the Morris water maze. (**C**) Retention performance, evaluated as the time spent in the light compartment following a single training session with an aversive thermal stimulus in the dark compartment. (**D**) Spontaneous alternation in Y-maze. Data are presented as mean ± SD (*n* = 6 per group). * *p* < 0.05 vs. control; # *p* < 0.05 vs. SCO.

SCO, a non-selective muscarinic acetylcholine receptor antagonist, disrupts cholinergic neurotransmission and produces reproducible impairments in learning and memory [7,8,28]. Spatial learning and memory were assessed using the Morris water maze test. The experimental schedule is shown in Figure 3A, and escape latency is presented in Figure 3B. SCO significantly increased escape latency compared with the no-treatment control, whereas KDC22-01 at 100 mg/kg significantly reduced escape latency compared with that induced by SCO. In the passive avoidance test, the time spent in the light compartment was significantly reduced in the SCO group compared with the control group (Figure 3C), indicating impaired retention memory. KDC22-01 treatment at 100 mg/kg significantly increased the time spent in the light compartment compared with SCO, whereas the positive effect of 300 mg/kg did not reach statistical significance. These results indicate that KDC22-01 at 100 mg/kg significantly attenuated scopolamine-induced impairment in passive avoidance performance under the present experimental conditions.

Short-term working memory was evaluated using the Y-maze test. In mice intraperitoneally injected with SCO, the spontaneous alternation ratio was significantly decreased compared with that in the control group. KDC22-01 treatment increased the spontaneous alternation ratio by 25% (100 mg/kg) and 31% (300 mg/kg) compared with SCO, respectively; however, these increases did not reach statistical significance (Figure 3D). Collectively, these findings indicate that KDC22-01 improved selected measures of SCO-induced cognitive impairment, with significant effects observed in the Morris water maze and passive avoidance test at 100 mg/kg, whereas the improvement in Y-maze performance did not reach statistical significance.

Similar improvements in SCO-induced cognitive impairment have been reported following the administration of natural product-derived extracts or phenolic compounds, as assessed using Morris water maze, passive avoidance, and Y-maze testing [29,30,31,32]. However, KDC22-01 did not significantly improve Y-maze performance, which may reflect differences among the three tests in the cognitive processes and neural substrates they assess. The Morris water maze evaluates hippocampus-dependent spatial learning and memory, in which mice use distal cues to locate a hidden platform under escape motivation [33]. The passive avoidance test measures aversively motivated associative memory acquired in a single trial; it depends on the hippocampus and amygdala and is assessed as retention of the aversive experience [34]. In contrast, spontaneous alternation in the Y-maze reflects short-term spatial working memory driven by exploratory behavior without reward or punishment; it depends on the hippocampus, septum and prefrontal cortex and is readily disrupted by muscarinic receptor blockade [34,35]. At the molecular level, spatial and associative long-term memories involve hippocampal synaptic plasticity and BDNF–TrkB–ERK/CREB signaling, which has been implicated in the memory-enhancing effects of *I. foliacea* extracts [14], whereas spontaneous alternation is thought to depend more directly on ongoing cholinergic transmission during testing [34]. Because a water extract of *I. foliacea* showed little acetylcholinesterase inhibitory activity in vitro [36], KDC22-01 may be less effective in counteracting the acute muscarinic blockade that impairs working memory than in supporting the hippocampal processes underlying spatial and associative memory. In addition, spontaneous alternation shows considerable inter-individual variability, and the small sample size (*n* = 6 per group) may have limited the power to detect the observed 25–31% improvements.

### 3.4. KDC22-01 Suppresses Hippocampal Tau Phosphorylation

**Figure 4:**
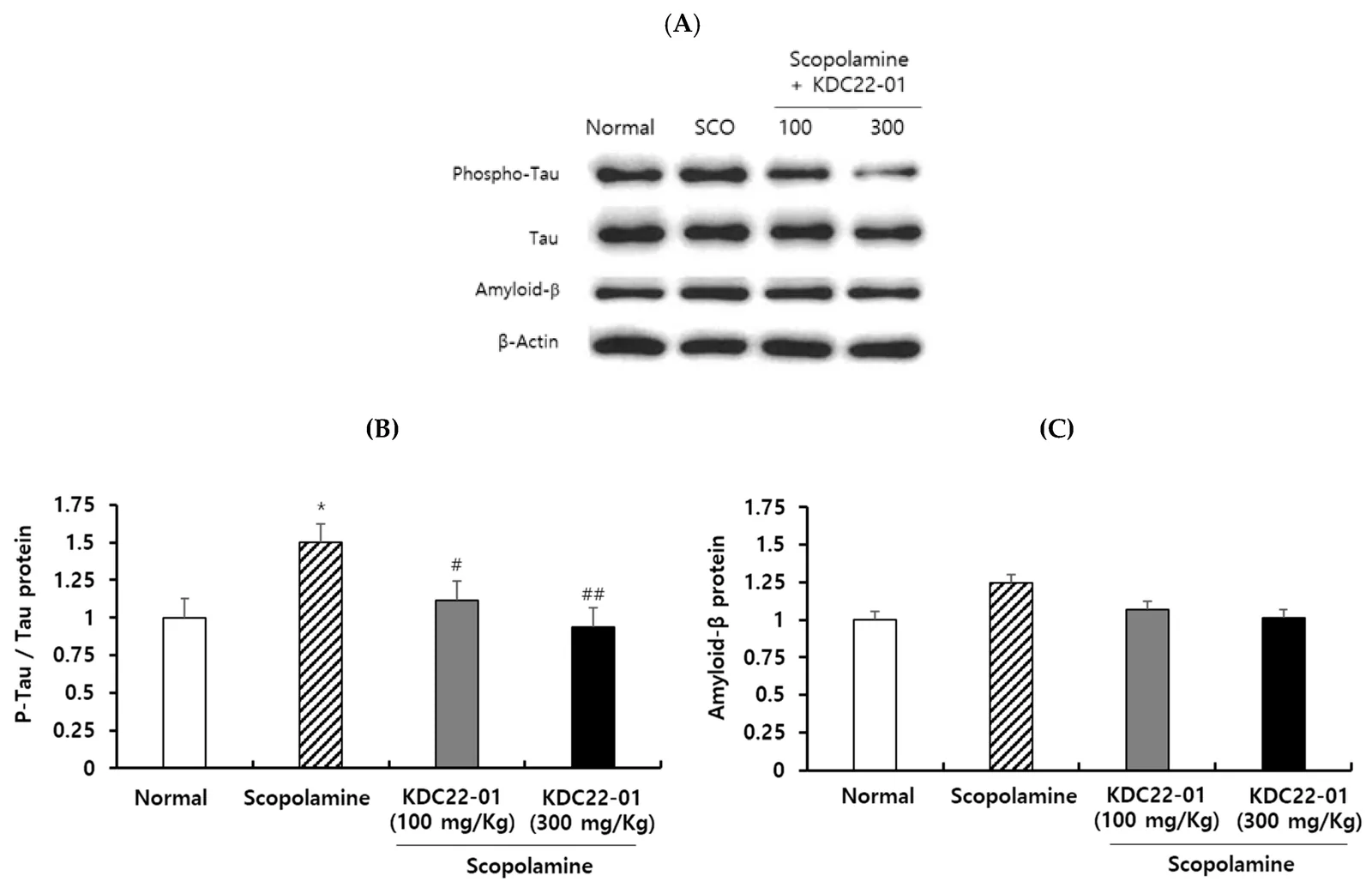
Effects of KDC22-01 on hippocampal tau phosphorylation and Aβ levels in SCO-treated mice. (**A**) Representative Western blot images of *p*-tau (Ser396), total tau and Aβ1–42 in hippocampal lysates from each group. β-Actin was used as a loading control. (**B**) *p*-Tau/total tau ratio derived from band intensities (mean ± SD, *n* = 6 per group). (**C**) Hippocampal Aβ1–42 levels normalized to β-actin, derived from band intensities. KDC22-01 treatment showed a suppressive, albeit non-significant, effect. * *p* < 0.01 vs. control; # *p* < 0.05, ## *p* < 0.01 vs. SCO.

To investigate the molecular changes associated with the behavioral effects of KDC22-01, we first examined hippocampal tau phosphorylation using Western blot analysis (Figure 4A). The *p*-tau (Ser396)/total tau ratio was significantly increased in the SCO group compared with the control group (Figure 4B). Treatment with KDC22-01 at both 100 and 300 mg/kg significantly reduced the SCO-associated increase in *p*-tau (Ser396). Ser396 is among the tau phosphorylation sites associated with pathological tau alterations in AD, although tau phosphorylation is highly site-dependent and heterogeneous across physiological and pathological conditions [37,38,39]. Therefore, the reduction in hippocampal *p*-tau Ser396 supports an effect of KDC22-01 on tau phosphorylation but does not establish a global reduction in pathological tau species. These findings indicate that KDC22-01 attenuated scopolamine-associated tau phosphorylation in the hippocampus. Hippocampal Aβ1–42 levels tended to increase in the SCO group (by approximately 25%) and to decrease with KDC22-01 treatment, but these changes were not statistically significant (Figure 4C). The more pronounced effect of SCO on tau phosphorylation than on Aβ1–42 levels may reflect differences in their regulation: tau phosphorylation is rapidly altered by kinase–phosphatase activity, and blockade of muscarinic M1 receptors disinhibits GSK-3β, whereas changes in Aβ require altered APP processing and accumulation over time and are less likely to be detected after short-term SCO treatment in wild-type mice [40]. Recent clinical evidence indicates that reducing cerebral Aβ burden can modestly slow down clinical decline in early AD, as demonstrated in phase 3 trials of lecanemab and donanemab, although amyloid removal alone does not halt disease progression [41,42]. In parallel, cerebral tau burden is closely associated with subsequent cognitive decline and progression of brain atrophy [6,43]. The concordant in vitro and in vivo findings suggest that modulation of tau phosphorylation may contribute to the neuroprotective effects of KDC22-01. However, because the SCO model represents acute cholinergic cognitive impairment rather than the progressive neuropathology of AD, these findings should not be interpreted as evidence of disease-modifying activity against AD.

Several study limitations should be considered. First, the SCO model represents acute pharmacological disruption of cholinergic neurotransmission and does not reproduce the progressive amyloid and tau pathology of AD. Second, the relatively small number of animals and the use of male mice only limit the generalizability of the behavioral findings. Third, the in vivo study included neither a pharmacological positive control, such as donepezil, nor a group treated with KDC22-01 alone without SCO. Therefore, the magnitude of the cognitive effect could not be directly benchmarked against a reference drug. In addition, although KDC22-01 alone did not affect cell viability in vitro (Figure 1C), its effects on behavior and hippocampal tau phosphorylation in mice without SCO remain to be determined. These controls should be included in future studies. Fourth, tau phosphorylation was assessed at a single site in each model: Ser199 in SH-SY5Y cells and Ser396 in the hippocampus. Although both sites are regulated by GSK-3β, CDK5 and PP2A [5,23], the use of different sites limits direct comparison between the in vitro and in vivo results, and other disease-relevant sites, such as Thr181, Thr217, Thr231 and Ser202/Thr205, were not examined. In addition, the upstream signaling mechanisms responsible for the reduction in tau phosphorylation, including the activities of GSK-3β, CDK5 and PP2A, were not assessed [37,38,39,44,45]. Future studies should evaluate a common panel of phosphorylation sites in both models. Moreover, each cell model was used for a single endpoint: tau phosphorylation was not measured in Aβ25–35-treated cells, and Aβ production was not measured in OA-treated cells. Future studies should examine whether KDC22-01 reduces Aβ-induced tau phosphorylation [19] and affects Aβ production, for example, in APP-overexpressing cells. Furthermore, Aβ1–42 was assessed by Western blot, which is less sensitive than immunoassays for the low levels of endogenous Aβ in wild-type mice; Aβ40 and Aβ42 should be quantified by ELISA in future studies. Fifth, although dieckol was identified as the marker phlorotannin in the HPLC fingerprint, the contents of dieckol and other individual phlorotannins in KDC22-01 were not quantified. Quantitative standardization of marker compounds will be required to ensure batch-to-batch consistency in the development of KDC22-01 as a functional ingredient.

## 4. Conclusions

In summary, KDC22-01, an aqueous extract of *I. foliacea*, exerted cytoprotective effects against pre-aggregated Aβ25–35-induced toxicity in SH-SY5Y cells and attenuated OA-induced tau hyperphosphorylation. In SCO-treated mice, KDC22-01 improved selected measures of spatial and contextual memory and significantly reduced hippocampal tau phosphorylation at Ser396. These findings suggest that modulation of tau phosphorylation may contribute to the cognitive protective effects of KDC22-01 and support further investigation of its underlying mechanisms and potential application as a functional-food ingredient.

## Data Availability

The data presented in this study are available upon request from the corresponding author. The data are not publicly available because they form part of an ongoing product development program.

## Supplementary Materials

The following supporting information can be downloaded at: https://www.mdpi.com/article/10.3390/nu18213517/s1, Figure S1: Representative HPLC chromatogram of KDC22-01 showing the marker compound dieckol.

## Abbreviations

The following abbreviations are used in this manuscript:

AD	Alzheimer’s disease
ANOVA	Analysis of variance
ELISA	Enzyme-linked immunosorbent assay
GAE	Gallic acid equivalents
OA	Okadaic acid
QE	Quercetin equivalents
PVDF	Polyvinylidene difluoride
SCO	Scopolamine
